# In China, Students in Crowded Dormitories with a Low Ventilation Rate Have More Common Colds: Evidence for Airborne Transmission

**DOI:** 10.1371/journal.pone.0027140

**Published:** 2011-11-16

**Authors:** Yuexia Sun, Zhigang Wang, Yufeng Zhang, Jan Sundell

**Affiliations:** 1 School of Environmental Science and Engineering, Tianjin University, Tianjin, China; 2 Architecture Engineering Department, Pennsylvania State University, State College, Pennsylvania, United States of America; 3 Tianjin Municipal Engineering Design and Research Institute, Tianjin, China; 4 The Faculty of Urban Construction and Environmental Engineering, Chongqing University, Chongqing, China; 5 School of Architecture, Tsinghua University, Beijing, China; University of Liverpool, United Kingdom

## Abstract

**Objective:**

To test whether the incidence of common colds among college students in China is associated with ventilation rates and crowdedness in dormitories.

**Methods:**

In Phase I of the study, a cross-sectional study, 3712 students living in 1569 dorm rooms in 13 buildings responded to a questionnaire about incidence and duration of common colds in the previous 12 months. In Phase II, air temperature, relative humidity and CO_2_ concentration were measured for 24 hours in 238 dorm rooms in 13 buildings, during both summer and winter. Out-to indoor air flow rates at night were calculated based on measured CO_2_ concentrations.

**Results:**

In Phase I, 10% of college students reported an incidence of more than 6 common colds in the previous 12 months, and 15% reported that each infection usually lasted for more than 2 weeks. Students in 6-person dorm rooms were about 2 times as likely to have an incidence of common colds ≥6 times per year and a duration ≥2 weeks, compared to students in 3-person rooms. In Phase II, 90% of the measured dorm rooms had an out-to indoor air flow rate less than the Chinese standard of 8.3 L/s per person during the heating season. There was a dose-response relationship between out-to indoor air flow rate per person in dorm rooms and the proportion of occupants with annual common cold infections ≥6 times. A mean ventilation rate of 5 L/(s•person) in dorm buildings was associated with 5% of self reported common cold ≥6 times, compared to 35% at 1 L/(s•person).

**Conclusion:**

Crowded dormitories with low out-to indoor airflow rates are associated with more respiratory infections among college students.

## Introduction

“Common cold” is a conventional term for a mild upper respiratory illness, with symptoms such as nasal blockage and discharge, sneezing, sore throat and cough [1]. Adults typically have 2–5 common colds per year, and children 4–8 colds [2]. Although such infections are often regarded as trivial, the cost to society is large [3]. Rhinoviruses have been associated with 40–65% of “common colds” through the year [4], and up to 80–92% of colds during outbreaks [5].

Cross-infection from an infected person to a healthy person depends on a number of factors, including how many viral particles are shed by the infected person, and the viral particles' survivability, both over time and with respect to distance from source in a shared environment. Three main mechanisms have been proposed for transmission of viruses causing airways infections:

- contact with secretions that contain the virus, either directly (e.g. hand to hand) from an infected person or indirectly from surfaces (e.g. door knob),
- “large” airborne droplets, which are produced by an infected person during talking, sneezing, or coughing, and can only spread in air for a distance of less than 1–2 m before falling down,
- “small” droplet nuclei (dried droplets), that can stay airborne for an extended time and be transported long distances.

Despite many years of study, the routes of spread of viral airways infections remain controversial. One opinion is that the virus is transferred through direct contact [6], while the other is that the virus is transferred through airborne spread [7], [8]. During the SARS epidemic, early preventive messages to the public were to wash hands and, generally to avoid “direct” contact spread. Later, analysis of the temporal and spatial distributions of SARS cases in a large community outbreak in Hong Kong and the correlation of these data with the three-dimensional spread of a virus-laden aerosol plume indicated an important role for airborne spread of droplet nuclei [9].

The influence of building characteristics including ventilation on the spread of viral respiratory infections has begun to receive increased attention from the public, government, media and scientists [10]. Brundage et al. [11] studied the risk of febrile acute respiratory diseases at four army training centers and found that disease rates were significantly higher among trainees in modern energy efficient barracks that had a low ventilation rate. Menzies et al. [12] suggested that there was a relationship between lower ventilation rates and more frequent tuberculosis infections among hospital workers. Milton et al. [13] reported an association between sick leave of employees and outdoor air supply rate. Myatt's [14] study showed that the probability of detecting airborne rhinoviruses was positively associated with weekly average CO_2_ concentration in an office. Other factors found to be associated with rate of infectious diseases include occupancy level [15], cleaning routines and “damp” buildings [16]. With respect to crowding, direct and surface contact as well as airborne transmission both appears to be factors in disease transmission. Hoge et al. found that severe overcrowding and inadequate ventilation contributed to an outbreak of pneumococcal disease in a large urban jail [17].

In China, one 20 m^2^ dormitory room is shared by 6–8 bachelor students or 4 master students or 3 PhD students. While such crowded spaces may be important sites for the propagation of respiratory infections, few studies have examined dorm room ventilation and its possible association with infection transmission.

The aim of this paper is to test whether the common cold is associated with how crowded a dorm room is and how well ventilated it is among college students in China.

## Methods

### Ethics statement

Verbal consents were obtained from participants, since participants did not want to be tracked back by signature. Both the study and the consent procedure were approved by the ethics committee at Tianjin University.

### Recruitment and measurement procedure

This study is part of the “Dorm Environment and Occupants' Health” study, which was carried out from 2006 to 2007 at Tianjin University, China. Details of the recruitment process and questionnaire contents have been previously described [18].

In brief, this study consisted of two phases. In Phase I, demographic information, the health status of 6500 students, and building and room characteristics of 2117 dorm rooms at Tianjin University were surveyed by questionnaires. The questionnaire survey was anonymous, but building number and room number were reported by participants. Project members visited dorm rooms, distributed questionnaires and explained to participants how to fill out questionnaires. The questionnaires were collected 2 days later. The questions on common cold infections were “how many times have you had a common cold in the previous 12 months (options: <6 times; 6–10 times; >10 times)” and “how long does a common cold usually last (options: <2 weeks; 2–4 weeks; >4 weeks)”. Other questions were about frequencies of window opening, cleaning routines and environmental tobacco smoke (ETS) exposure.

In Phase II, air temperature, relative humidity and CO_2_ concentration in dorm rooms were measured by indoor air quality monitor PS 31 (http://www.sensotron.pl) for 24 hours. Air quality monitors were calibrated at the International Center for Indoor Environment and Energy, Technical University of Denmark prior to measurements. Dorm occupants reported opening status of doors and windows at day and at night during measurement (options: completely close; 2 cm open; 5 cm open; 50% open; completely open).

The out-to indoor air flow rate at night was calculated from an analysis of the build-up period of metabolic CO_2_ produced by sleeping occupants (1:00 a.m.–8:00 a.m.) [19]. Calculation details are described in Information S1. CO_2_ concentrations of dorm rooms were measured both in the summer (May–Jul., 2006) and in the winter (Dec., 2006–Apr., 2007) [20]. The average indoor air temperature and relative humidity at night were calculated (1:00 a.m.–8:00 a.m.). Outdoor CO_2_ concentration and meteorological parameters were also measured on campus during the same time.

### Statistics

The associations among gender, age, whether family member ever had asthma and allergy, environmental tobacco smoke, cleaning routine, window opening frequency, occupancy levels, and self-reported common cold incidence and duration were analyzed by Chi-square tests. Adjusted odds ratios of crowdedness and air flow rate for common cold infections were evaluated in multiple logistic regression models. A carbon dioxide-based risk equation [21] was used to calculate the basic reproductive number of common colds which was compared to the self-reported infection rate.

A *P* value less than 0.05 indicates statistical significance. SPSS software 15.0 was used to perform the statistical analyses.

## Results

### Phase I

In Phase I, 3712 students living in 1569 dorm rooms in 13 buildings answered the questionnaire, giving a response rate of 57%. Surveys for 276 students were excluded from the analysis due to missing information. Forty eight percent (48%) of students were female. PhD students' mean age was 29 years, master students 25 years and bachelor students 22 years. Monday through Friday, 18% of participants spent less than 2 hours indoors watching TV/playing games per day, 36% spent 2–10 hours per day, and 46% spent more than 10 hours per day. Dorm buildings had 3–12 floors, with 26–43 dorm rooms per floor. All floors in each dorm building are homogeneous with regard to occupants' gender and education level. Dorm rooms consisted of one simple bedroom. Each floor provided two washing rooms and restrooms. Six bachelor students, 4 master students or 3 PhD students shared one dorm room with a volume of 50–70 m^3^. The average density was 5 m^2^ per person. Based on the questionnaire data from Phase I, 238 dorm rooms with 473 students living in these dorms were evaluated for Phase II. The evaluated dorm rooms represented different building structures, construction periods, locations and occupancy levels. There were no significant differences in students' ages, gender, self-reported common cold incidence or duration between Phase I and Phase II.

In the questionnaire survey of Phase I, 249 out of 3436 (7.3%) students reported 6–10 common colds in the previous 12 months, while 94 (2.8%) reported more than 10 common colds. Four hundred and thirty six (12.8%) students had common colds lasting for 2–4 weeks, while 65 (1.9%) reported colds lasting more than 4 weeks. Demographic information and living habits of dormitory occupants, and their associations with common cold are summarized in Table 1. Atopy was associated with increased incidence and longer duration of common cold. Male students were more susceptible than females, but had shorter duration colds. Females cleaned rooms more often than males, cleaning rooms at least twice per week 52% compared to 31% for males, and smoked less (1.4% vs. 15.9%). Passive smoking had a significant effect on the incidence of common cold (p = 0.029), but after adjustment for environmental tobacco smoke, males were still at greater risk for common colds (p = 0.010). Younger students lived in more crowded rooms and reported longer duration colds. Crowding, not age, was shown by stratification for occupancy level to be the significant association with common cold duration.

**Table 1:** Associations between common cold and demographic information and living habits of 3436 dormitory occupants, 2006–2007.

	Number	Percent, %							
		Common cold incidence				Common cold duration			
Total	3436	< 6 times	6-10 times	> 10 times	p^3^	<2 wks	2-4 wks	>4 wks	p
Gender									
Male	1782	88.3	8.5	3.2		87.7	10.6	1.8	
Female	1654	91.7	6.0	2.3	**0.006**	82.7	15.2	2.1	**0.000**
Age									
≤23 yrs	2012	89.9	7.8	2.2		84.0	14.2	1.8	
24-26 yrs	715	92.1	5.3	2.5		88.6	10.3	1.1	
≥27 yrs	219	92.6	5.1	2.3	0.164	90.3	8.3	1.4	**0.009**
Atopy 1									
Yes	231	79.5	15.3	5.2		68.9	26.3	4.8	
No	3120	90.7	6.8	2.6	**0.000**	86.5	11.8	1.7	**0.000**
ETS 2									
Yes	573	87.4	10.0	2.6		86.3	10.9	2.8	
No	2799	90.3	6.8	2.8	**0.029**	85.0	13.2	1.8	0.098
Cleaning routine									
Every day	509	91.3	6.5	2.2		85.2	13.4	1.4	
1-2 times/week	1844	90.8	6.7	2.5		85.4	12.8	1.8	
<2 times/week	1034	87.8	8.6	3.6	0.088	84.8	12.7	2.4	0.644
Opening window									
Every day	2816	90.2	7.0	2.8		85.0	13.1	1.9	
1-2 times/week	467	88.8	8.0	3.2		85.8	12.6	1.5	
<2 times/week	107	87.7	10.4	1.9	0.598	87.6	8.6	3.8	0.397

1 ^.^ Whether any family member ever had asthma and allergy.

2 ^.^ Environmental tobacco smoke.

3 ^.^ Pearson Chi-square test.

**Figure 1:**
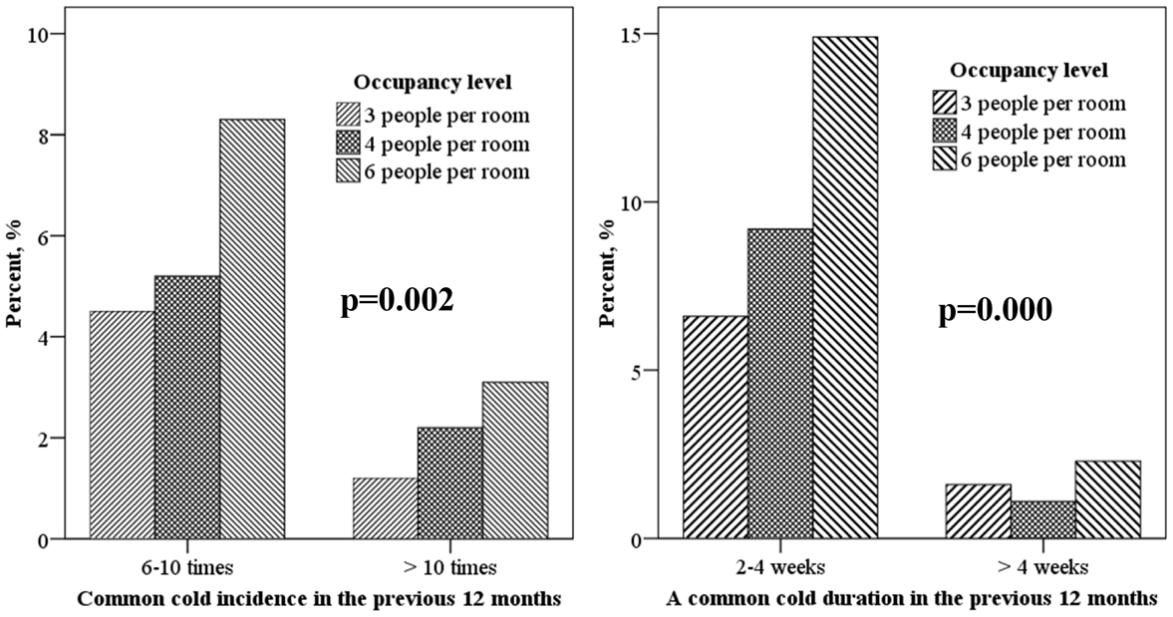
Comparison of common cold incidence and duration for different occupancy levels.

**Figure 2:**
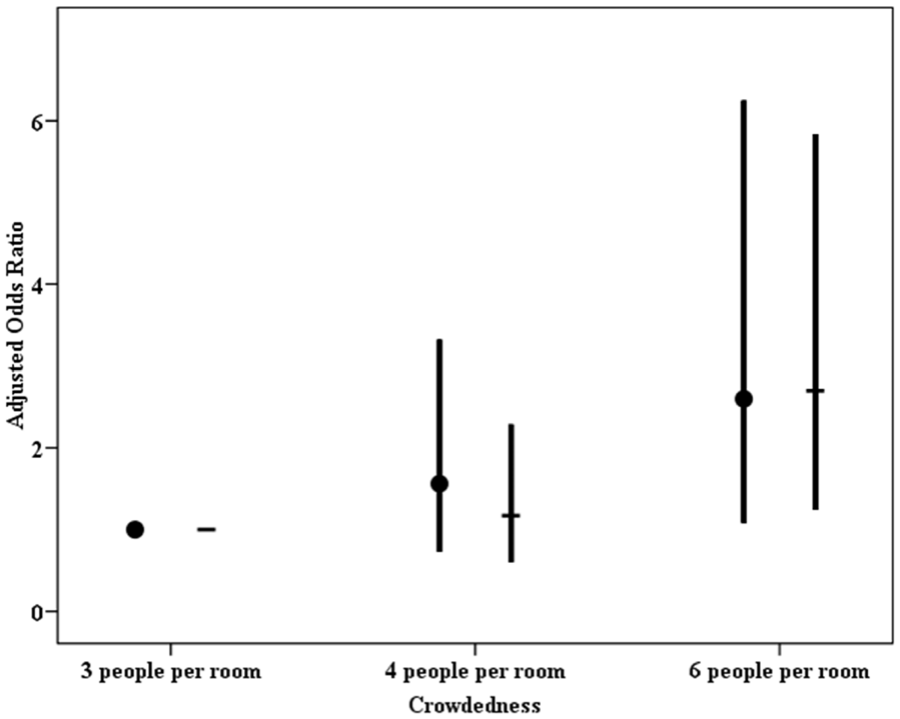
Associations between crowdedness and common cold annual incidence ≥6 times and duration ≥2 weeks. Odds ratios were adjusted for gender, age, hours spent indoors, family member allergy history and exposure to environmental tobacco smoke. Circles represent adjusted odds ratio (AOR) for incidence. Dashes represent AOR for duration. 95% confidence interval is demonstrated.

Self-reported common cold incidence and duration are compared for different occupancy levels in Figure 1. With incrementally increasing occupancy in dorm rooms, the proportion of occupants with ≥6 common colds increased significantly (p = 0.002), as did the proportion of occupants with ≥2 weeks common cold duration (p = 0.000). The odds ratios of crowdedness for common cold incidence of ≥6 times and duration of ≥2 weeks, adjusted for gender, age, hours spent indoors, family members' asthma and allergy history, environmental tobacco smoke exposure are shown in Figure 2. Students in 6-person rooms were about 2.0 times as likely to have a common cold incidence ≥6 times per year and a duration ≥2 weeks, as students in 3-person dorm rooms.

### Phase II

For Phase II, the evaluated dorm rooms were located in 13 buildings. Four were built between 1940 and 1960, two between 1977 and 1983, three between 1993 and 1999, and four after 2000. For newly constructed dorm buildings, concrete structure and PVC frame windows were used instead of the brick-stone structure and the wooden frame windows used in older buildings. Ventilation for all dorm rooms consisted solely of opening doors and windows. The out-to indoor air flow rates for rooms measured during summer varied significantly, from 0.8 to 110 L/s per person, with a median of 18 L/s per person. Air flow rates measured in the heating season (from Dec. 5, 2006 to Apr. 14, 2007) varied from 0.3 to 24 L/s per person, with a median of 3.0 L/s per person. Ninety percent of the dorm rooms had an out-to indoor air flow rate less than 8.3 L/s per person.

The average indoor air temperature (mean 28.0°C, 95% confidence interval (CI) 27.8°C–28.3°C, range 22.0°C–32.1°C) and relative humidity (mean 54%, 95% CI 53%–55%, range 27%–78%) in summer were high and had large variations consequent to opening doors and windows as the sole mode of ventilation. During the winter season when the heating system was in use and doors and windows were closed, weather conditions had less influence on the indoor thermal environment (temperature: mean 21.0°C, 95% CI 20.7°C–21.3°C, range 15.4°C–26.5°C; relative humidity: mean 40%, 95% CI 38%–41%, range 18%–72%). Data for temperature and relative humidity in rooms with different occupancy levels and out-to indoor air flow rates are shown in Table 2. In summer, relative humidity and temperature were not different in rooms with different air flow rates. An inverse association between occupancy level and relative humidity was caused by the measurement sequence (6-person dorms were measured at the driest time in May, whereas 3-person dorm rooms were measured in July when outdoor relative humidity was higher). Outdoor climate is the dominating factor in determining the indoor temperature and relative humidity in summer. In winter, rooms shared by 6 people had the highest relative humidity and temperature at night. A low out-to indoor air flow rate was related to a significantly higher relative humidity (p = 0.000). However, common cold infections were not significantly associated with indoor air temperature (p = 0.806) and relative humidity (p = 0.642).

**Table 2:** Temperature and relative humidity in rooms with different occupancy levels and outdoor air flow rates, mean (standard deviation).

		Temperature, °C	Relative humidity, %
Summer	Occupancy level		
	3 people per room	29.6 (1.1)	64 (8)
	4 people per room	28.4 (2.0)	55 (8)
	6 people per room	27.1 (2.1)	49 (8)
	Out-to indoor air flow rate		
	Above median (18 L/s per person)	28.6 (1.9)	54 (10)
	Below median	27.5 (2.2)	53 (10)
Winter	Occupancy level		
	3 people per room	20.4 (2.3)	39 (11)
	4 people per room	20.8 (2.5)	36 (8)
	6 people per room	21.8 (2.1)	43 (9)
	Out-to indoor air flow rate		
	Above median (3.0 L/s per person)	20.8 (2.2)	34 (7)
	Below median	21.4 (2.3)	45 (8)

**Figure 3:**
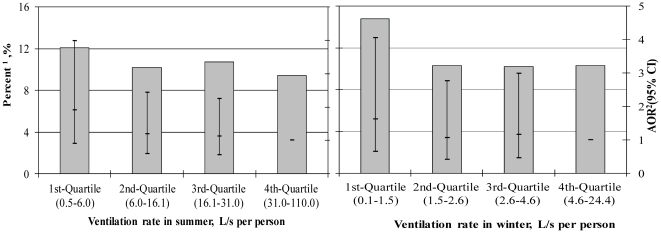
Associations between ventilation rate and common cold annual incidence ≥6 times. ^1^ Proportion of occupants with ≥6 common colds in the previous 12 months. ^2^ Odds ratios were adjusted for gender, age, family member allergy history, exposure to environmental tobacco smoke, building age and crowdedness. AOR: adjusted odds ratio; CI: confidence interval.

Figure 3 shows that the lowest quartile of out-to indoor air flow rates per person in both summer and winter were associated with an increased proportion of occupants with ≥6 common colds in the previous 12 months. The adjusted odds ratios of ventilation rates for common cold infections increased slightly across the quartiles. The critical ventilation rate, below which common cold incidence increases, is identified. When ventilation rate is below 6 L/s per person, the common cold incidence in dorm rooms with average 4 occupants increased from 10% to 12%. When ventilation rate is below 1 L/s per person, the common cold incidence increased from 10% to 15%.

**Figure 4:**
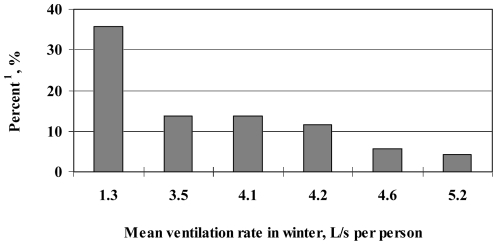
Associations between common cold infection rates and mean ventilation rate in winter in buildings constructed after year 1993. ^1^ Proportion of occupants with ≥6 common colds in the previous 12 months.

In our study, old buildings had more dampness problems, while new buildings using modern construction technologies had smaller ventilation rates [21]. Dampness problems have been reported to be associated with an increased incidence of common cold infections [18]. In order to eliminate the influence of indoor environmental factors other than poor ventilation, the mean ventilation rates in newly constructed dorm buildings were calculated and related to the percentage of occupants with common cold infections more than 6 times annually. The ventilation rates in winter are less than those in summer, and may help nail down the critical ventilation rate, below which common cold incidence increases. Figure 4 shows that the infection rate of common colds in the “tight” buildings constructed after 1993 is, in winter, associated with mean ventilation rate. There were 7 buildings constructed after 1993. One building was not included in the analysis because measurements were performed in only 9 dorm rooms. On average, there were 1140 occupants in each dorm building. A mean ventilation rate of 5 L/(s•person) was associated with ≥6 common colds per year in 5% of occupants , compared to a 35% for 1 L/(s•person). There were 6 buildings constructed before 1993, among which 4 buildings had <10 dorm rooms measured in winter and were excluded from the analysis. Of the remaining 2 buildings, one had mean ventilation rate of 5.7 L/(s•person) and a common cold infection rate of 23.8%, while the other had a mean ventilation rate of 6.4 L/(s•person) and a common cold infection rate of 7.1%.

### CO_2_-based risk equation

The Wells-Riley equation estimates the number of secondary infections that arise when a single infectious case is introduced into a population where everyone is susceptible [22]. This number is called the basic reproduction number. Rudnich and Milton [23] expanded the Wells-Riley equation to apply to situations with non-steady state conditions and variable ventilation rates:
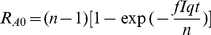
(1)Where R_A0_ is the basic reproduction number; n is the number of occupants; f is the re-breathed fraction; and I is the number of infectors ( = 1). q is the quantum generation rate by an infected person (quanta/h), where a quantum is the amount of infectious material needed to produce infection in 63% of uniformly exposed animals, and is therefore 1.25 times the median infectious dose, 1.25×ID_50_. t is the exposure time (h); f = (C-C_0_)/C_a_, where C_a_ is the volume fraction of CO_2_ added to exhaled breath, C is the volume fraction of CO_2_ in indoor air, and C_0_ is the volume fraction of CO_2_ in outdoor air.

The incidences (<6 times; 6–10 times; >10 times) and durations (<2 weeks; 2-4 weeks; >4 weeks) of common colds in the previous 12 months for different occupancy levels (6-people; 4-people; 3-people per dorm) were self-reported by occupants. The mean duration of a common cold is 7–10 days [1]. For this study we assumed that the duration of a common cold was 9 days. Although many viruses can produce symptoms of common cold, rhinovirus is the most frequent cause of the common cold [24]. Riley and Nardell suggested that q for rhinovirus is in the range of 1–10/h [25]. Here we inferred q = 9/h. We assumed that the infector remained in the dorm room 8 hours per day. The average CO_2_ concentrations in each dorm room from 1:00 a.m. to 8:00 a.m. were calculated. The estimated and self-reported number of common colds in each day in winter is compared (Table 3). These two numbers fit very well indicating the validity of this CO_2_-based risk model in predicting infection rate of infectious disease like common cold.

**Table 3:** Comparison of estimated and self-reported basic reproduction number of common cold per day.

		Percentage of students with self-reported common cold incidence			Indoor CO_2_ concentration (C)	Re-breathed fraction (f)	Basic reproduction number of common cold	
		<6 times	6-10 times	> 10 times			Self-reported (R_A0_)	Estimated (R_A0_’)
Occupancy level (O)	6 (O_6_)	88.6 (D_16_)	8.3 (D_26_)	3.1 (D_36_)	1483 (C_6_)	0.032 (f_6_)	1.6	1.6
	4 (O_4_)	92.6 (D_14_)	5.2 (D_24_)	2.2 (D_34_)	1021 (C_4_)	0.020 (f_4_)	1.0	0.9
	3 (O_3_)	94.2 (D_13_)	4.5 (D_23_)	1.2 (D_33_)	1011 (C_3_)	0.019 (f_3_)	0.7	0.7

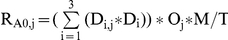
, person/day.

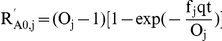
, person/day.

D_i_ is the assumed number of common cold infections in winter under different self-reported incidence rate, times. i indicates common cold incidence. i = 1, 2, 3. 1-common cold less than 6 times in the previous 12 months; 2-common cold 6-10 times; 3-comon cold more than 10 times. We assume D_1_ = 3; D_2_ = 6; D_3_ = 8.

O_j_ is the occupancy level, person/room. j indicates occupancy level. j = 3, 4, 6. 3-three people per dorm room; 4-four people per dorm room; 6-six people per dorm room.

D_i,j_ is the proportion of students with different self-reported common cold incidences, %.

M is the duration of a common cold, days. We assume M = 9 days [1].

T is days in winter season, 120 days.

C_j_ is the average CO_2_ concentration from 1:00 a.m. to 8:00 a.m. in rooms with different occupancy levels, ppm.

f_j_ is the re-breathed fraction of indoor air in rooms with different occupancy levels. f_j_ = (C_j_-C_0_)/C_a_. C_a_ is the volume fraction of CO_2_ added to exhaled breath, 37000 ppm. C_0_ is the volume fraction of CO_2_ in outdoor air, 300 ppm.

q is the quantum generation rate by an infected person, quanta/h. We assume q = 9 quanta/h [25].

t is the time a infector remaining in the dorm room, hour/day. We assume t = 8 hours per day.

If for a given population and infectious agent, the basic reproductive number >1 then that agent can spread in the population. The critical re-breathed fraction (f_c_), corresponding to a basic reproduction number of 1, can be derived from Equation (1), 
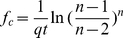
(2)


**Figure 5:**
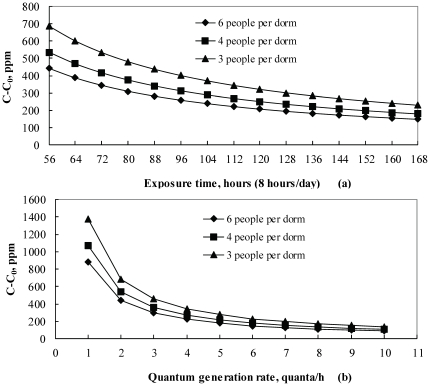
Critical indoor CO_2_ concentrations above background in dorm room as a function of exposure time and quantum generation rate. (a) Quantum generation rate  = 2 quanta/h, C_a_ = 37000ppm. (b) t = 56 hours (i.e. 7days), C_a_ = 37000 ppm.

In the present study, the critical re-breathed fraction in rooms with different occupancy levels and the associated critical indoor CO_2_ concentrations above background (outdoor CO_2_ concentration) were calculated from Equation (2), both as a function of exposure time (Figure 5(a)) and quantum generation rate (Figure 5(b)). Thus Figure 5 predicts the critical indoor CO_2_ concentrations beyond which infectious disease will spread. The family of curves in Figure 5(a) describes the trends of the critical indoor CO_2_ concentrations above outdoor values (C-C_0_) as a function of exposure times for risk of respiratory infections. The quantum generation rate used was 2/h. The critical CO_2_ concentration above the background levels off if the common cold lasts more than 3 weeks (exposure time 8 hours/day, totally 168 hours) (Fig 5(a)). This indicates that even for less infectious agents with quanta generation rate no more than 2/h, a full fresh outdoor air system without recirculation of indoor air needs to be used in environments where people spend extended time (for example bedrooms, dorms, schools, daycare centers) in order to prevent viral infections. In Figure 5(b), the exposure time was assumed to be 56 hours (8 hours/day, i.e. 7 days). It shows that the current ASHRAE standard of 700 ppm above the background level [26] would not prevent the infection from being spread in a dorm room with 6 occupants unless the quantum generation rate of infectious agents is no more than 1 quantum/h (Fig. 5(b)).

## Discussion

The campus living style and dormitory conditions of students at Tianjin University is typical of China. The sample size in our study is large, and the response rate was reasonably good (57%). No significant difference was found between respondents and non-respondents in reporting wheeze and dorm room dampness [18]. Thus it is highly unlikely that selection bias impacted the findings of this study. Common cold is a conventional term for a mild upper respiratory illness. College students can be expected to understand what “common cold” refers to. There is no evidence to suggest that bachelor students have a different memory in reporting common cold infection, compared to PhD students. Compared to home environment, dorm buildings are perceived to be very crowded no matter whether 3 or 4 or 6 people share a 20 m^2^ room. Even students in 3-people-shared dormitory think their space is crowded. Therefore, the significant association between occupancy level and incidence of common colds, and the dose-response relationship between ventilation rate and incidence of common colds cannot be explained by reporting bias.

The occupants' education level was not adjusted for when calculating the odds ratios of crowdedness for common cold infections since 3 PhD students or 4 master students or 6 bachelor students share one dorm room with similar volume. Education level itself should not be a confounding factor. Psychological stress, related to education status may have effect on common cold as indicated in a previous study [27]. However, our study found that less crowded dorm rooms occupied by PhD students were associated with less common cold infections. This cannot be explained by psychological stress since PhD students are supposed to be more stressed than master or bachelor students.

The summer measurement was from May to July and winter measurement from December to April. In summer measurements, 6-people-shared dormitories were measured first, followed by 4 or 3 people shared dormitories. In winter measurement, dorm buildings were measured randomly. There could be a potential systematic bias for summer measurement, but not for winter measurements. During the measurements, outdoor CO_2_ concentrations and meteorological parameters were monitored. In principle, air change rate in buildings with natural ventilation system is not influenced by air relative humidity. Outdoor air temperature itself and the consequent occupants' behavior (e.g. opening doors/windows) may influence the air change rate in dorm rooms. In our study, the opening of doors/windows was reported by occupants themselves. In winter, occupants tended to close doors and windows tightly, so that variations in winter outdoor temperature had little influence on ventilation rate in dorm buildings. In summer, the mean outdoor air temperature was 29.6°C, ranging from 22.5°C to 35.2°C. The median air change rate was 4.42 h^−1^ and 4.67 h^−1^ when outdoor air temperature is below and above 29.6°C. There was no significant difference of air change rate for different temperatures in summer (p = 0.319). Therefore, it is reasonable to assume that, the air change rates measured in summer and winter are representative for respective season, without influence from small climate changes within each period.

While it is possible that some of the self-reported common colds were influenza, the infection rate of flu among adults is approximately once per year in this part of China. Therefore, this possible error would not change our results. Moreover, common colds and influenza are spread in a similar way; the present study could have been titled “airways infections”. In each dorm room, CO_2_ concentrations were measured for 24 hours in both summer and winter. As measurements were made over a long period, i.e. summer measurements between May and July and winter measurements between December and April, and for 238 rooms, the mean values of ventilation rates should be valid for rooms with different occupancies and opening status of windows/doors, and for changes in the outdoor climate.

There were imperfections in our data collection. In some rooms occupants may have had the window open during the night measurements in winter. Perhaps the incidence of common cold was influenced by an influenza epidemic. These sources of error would shift our findings towards the null hypothesis, that there was no association between common cold infections and dorm crowdedness or ventilation rate. Our findings are robust in spite of these possible problems. Thus, it is likely that more measurements and more accurate data on types of airways infections would show an even stronger association.

The out-to indoor air flow rate required by the Indoor Air Quality Standard of China is 8.3 L/s per person [28]. In the present study, 90% of the dorm rooms measured during winter had night-time ventilation rates less than this value. CO_2_ concentration in corridors was not measured, so that the fresh out-to indoor air flow rate may have been even lower than the calculated value in cases when corridor windows were closed.

The suggested dose-response relationship between dorm ventilation rate and common cold infections among occupants can be extrapolated to other crowded public premises with substandard ventilation rate, meaning a possible important public health topic for e.g. schools, daycare centers.

Although it is widely held that people in crowded spaces have more airways infections [15], [29], there are few studies on this. Our study is among the first published suggesting a relationship between occupancy levels, ventilation rates, and respiratory infections. With 6 occupants instead of 3 in a 20 m^2^ dorm room, the proportion of occupants with incidence of more than 6 common colds in the previous 12 months doubled. When crowdedness is adjusted for, a lower ventilation rate is associated with an increased risk of common cold. This finding is consistent with Shendell's study in schools, which showed that a 1000 ppm increase in dCO_2_ (difference between indoor and outdoor CO_2_ levels) was associated with a 0.5%–0.9% decrease in annual average daily attendance [30]. For office buildings, Milton found that short-term sick leave was reduced by 35% at 24 L/s per person compared to 12 L/s per person outdoor air flow [13].

A crucial question is whether the increased frequency of common colds in crowded places is due to direct contact (or via surfaces), via droplets or via droplet nuclei. The strong association with ventilation in this study indicates that airborne transmission is important and perhaps the main route.

### Conclusion

Crowdedness and outdoor air ventilation per person are important for the spread of airborne infectious diseases in rooms such as dorms where people spend a lot of time. Respiratory viruses can be transmitted through air so that transmission is modulated by outdoor air supply rates. Further studies are warranted.

## Supporting Information

Information S1 **Dormitory outdoor air flow rate calculation by using CO_2_ method.**
(DOCX)
