# Supplementary material for: In China, Students in Crowded Dormitories with a Low Ventilation Rate Have More Common Colds: Evidence for Airborne Transmission
Source: PLoS One. 2011 Nov 16;6(11):e27140. doi: 10.1371/journal.pone.0027140 (PMC3217956; doi:10.1371/journal.pone.0027140)
Supplement: Information S1 — Dormitory outdoor air flow rate calculation by using CO2 method. (DOCX) [file pone.0027140.s001.docx]

**Dormitory outdoor air flow rate calculation by using CO_2_ method**

Each dorm room is one simple bedroom, which can be treated as a single zone. The individual step increase of CO_2_ concentration (Δc) between two measurements performed with time interval of Δτ in a point is described by the instantaneous flow rate equation as follows,

$\Delta c=\frac{\Delta\tau}{V_{zone}}\cdot[F-0.28Q\cdot(c_{1}-c_{out})]$ (1)

Where,

F is the emission rate of CO_2_ (m^3^/s), which depends on the level of activity (M, MET), height (H, m), weight (W, kg) and respiratory quotient (R, 0.83) of occupants.

$F=R\frac{0.00056028\cdot H^{0.725}\cdot W^{0.425}\cdot M}{0.23\cdot R+0.77}$ (2)

Q is the out-to indoor air flow rate (L/s);

c_1_ is the CO_2_ concentration measured at the beginning of the time interval Δτ (ppm);

c_out_ is the outdoor CO_2_ concentration (ppm).

The CO_2_ concentration at the end of the time interval (c_2_) can be computed as a sum of the initial concentration (c_1_) and the unit increase of concentration (Δc). The calculation can be repeated for each successive time interval so that a theoretical exponential curve can be constructed. This theoretical curve is fitted to the measured data using least squares. For this, the production rate of CO_2_ is set within a range of minimum and maximum values based on the weights and heights of occupants. This iterating process gives the best fitting air flow and emission rates for CO_2_ are found.
